# The Significance of iron deficiency and anemia in a real-life COPD cohort

**DOI:** 10.7150/ijms.46163

**Published:** 2020-08-19

**Authors:** Alex Pizzini, Magdalena Aichner, Thomas Sonnweber, Ivan Tancevski, Günter Weiss, Judith Löffler-Ragg

**Affiliations:** 1 Department of Internal Medicine II, Infectious Diseases, Pneumology, Rheumatology, Medical University of Innsbruck, Innsbruck, Austria.; 2 Christian Doppler Laboratory for Iron Metabolism and Anemia Research, Medical University of Innsbruck, Austria.

**Keywords:** COPD, obstructive pulmonary disease, anemia, iron deficiency, inflammation

## Abstract

**Background:** Current evidence suggests an increased prevalence of iron deficiency (ID) and anemia in chronic obstructive pulmonary disease (COPD). ID and subsequent anemia can be due to iron losses via bleeding resulting in absolute ID or inflammation-driven retention of iron within macrophages resulting in functional ID and anemia of inflammation.

**Methods:** This is a retrospective analysis of 204 non-exacerbated COPD patients in outpatient care. Current definitions of absolute and functional ID were applied to determine the prevalence of ID and to analyze associations to disease severity in terms of lung function parameters and clinical symptoms.

**Results:** The studied cohort of COPD patients demonstrated a high prevalence of ID, ranging from 30 to 40% during the observation time. At the initial presentation, absolute or functional ID was found in 9.3% to 12.3% of COPD individuals, whereas combined forms of absolute and functional ID were most prevalent (25.9% of all individuals). The prevalence of ID increased during longitudinal follow-up (37 ± 15 months), and especially combined forms of ID were significantly related to anemia. Anemia prevalence ranged between 14.2% and 20.8% during the observation period and anemia was associated with lower FEV1, DLCOc, and CRP elevation. Accordingly, ID was associated with decreased FEV1, DLCOc, and an elevation in CRP.

**Conclusion:** ID is common in COPD patients, but a uniform definition for accurate diagnosis does not exist. Prevalence of functional ID and anemia increased during follow-up. The associations of ID and anemia with reduced functional lung capacity and elevated inflammation may reflect a more severe COPD phenotype.

## Introduction

Chronic obstructive pulmonary disease (COPD) is the third leading cause of death worldwide, resulting in 3.0 million deaths in 2016 1. The main pathogenetic features of COPD are oxidative stress and protease/antiprotease imbalance leading to local airway inflammation and remodeling as well as chronic systemic inflammation 2-4.

Iron is a crucial micronutrient as it is involved in several metabolic processes, such as DNA synthesis, oxygen transport, cellular metabolism, and mitochondrial respiration 5, 6. On the other hand, environmental sources of iron and other particles can disrupt and interfere with local iron-homeostasis in the lung 7. Genes related to iron-metabolism are associated with COPD, and exposure to tobacco smoking, air pollution, and other harmful substances impact on regulatory mechanisms, potentially driving the pathogenesis of COPD 7-9. However, these processes are not limited to the lung, as inflammatory cytokines that are induced and released in the course of COPD 10, 11 can also impact iron homeostasis. Thereby, inflammatory cytokines and the increased expression of the master regulator of iron homeostasis hepcidin result in increased acquisition and storage of iron within cells of the reticuloendothelial system. This results in functional iron deficiency (FID) and anemia of inflammation (AI), reflected by low circulating iron levels and thus reduced availability of the metal for erythropoietic cells, whereas levels of the iron storage protein ferritin are normal or increased as a consequence of reticuloendothelial iron retention 11.

In contrast, true or absolute iron deficiency (AID) and subsequent iron deficiency anemia (IDA) occurs as a consequence of chronic blood losses and/or insufficient dietary iron absorption 12. Thus, low circulating iron and ferritin levels characterize this most frequent type of anemia. Besides, combined forms of AI and IDA (CID) can occur in patients with inflammation-driven iron retention and chronic gastrointestinal or urogenital blood losses 13. The identification of such patients is challenging because no single parameter can differentiate between AI and AI with combined IDA but of significant clinical interest. Patients with IDA and patients with combined AI/IDA benefit from iron supplementation. In contrast, its benefit-risk assessment has not been established in prospective trials in patients with FID, as it may be hampered by ongoing inflammation 11. Several clinical markers have been used to differentiate between AI and AI/IDA, including the soluble transferrin receptor (sTfR) reflecting the needs of iron for erythropoiesis 14-16. As it is also affected by inflammation, a ratio of sTfR/log ferritin was introduced, which should better differentiate between AI and AI/IDA 17. However, some overlap, especially in patients with advanced inflammation, remains 16, 18.

This retrospective study aimed to identify the prevalence of ID with/without concomitant anemia by applying robust and easily reproducible definitions for different types of ID and anemia in a real-life COPD cohort and to evaluate its impact on disease course.

## Methods

### Patients selection

**Figure 1:**
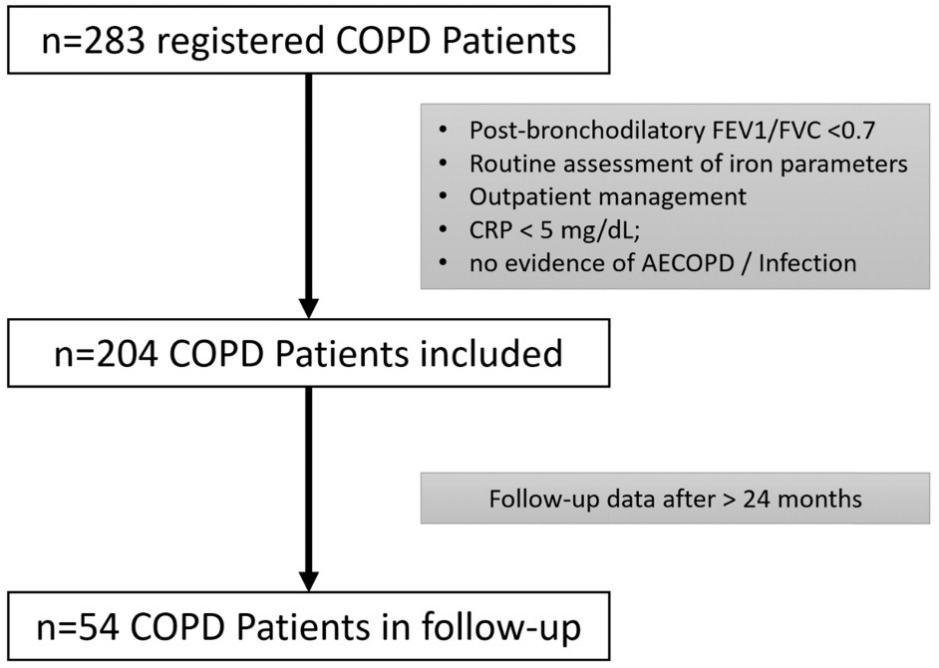
Algorithm of patient selection.

This is a single-center cohort study, including 204 COPD patients based on the COPD registry of the Pulmonary Outpatient Clinic of the Department of Internal Medicine II Innsbruck. Patients who were in routine treatment at the Pulmonary Outpatient Clinic between July 2007 and May 2017 were retrospectively recruited. All COPD patients were screened and included if the diagnostic criteria according to the Global Initiative for Chronic Obstructive Lung Disease (GOLD) guidelines 19, i.e., a post-bronchodilator fixed ratio of forced expiratory volume in one second (FEV1) versus forced vital capacity (FVC) less than 0.7 and COPD-typical symptoms were met. Patients in outpatient care with no evidence of acute infection or exacerbation (AECOPD) and only low-grade inflammation (CRP < 5mg/dl) were eligible. Further, only patients with available laboratory blood tests, including iron parameters on the day of lung function testing were included. Follow-up lung function tests and laboratory parameters were admitted if available from at least 24 months later. Patients were followed up as part of routine clinical management at the institution, either because they are not in treatment at an out-of-hospital licensed pulmonologist or have conditions requiring a specialized outpatient clinic. **Figure 1** shows the algorithm of patient selection.

Parameters related to COPD like spirometry, diffusion capacity for carbon monoxide corrected for hemoglobin (DLCOc), COPD assessment test (CAT) results, the frequency of severe acute exacerbations requiring hospitalization, and routine laboratory parameters were included in this analysis. Gastrointestinal comorbidities and anticoagulation therapy (ACT), defined as vitamin - k antagonists, factor-Xa inhibitors, low-molecular-weight heparin, and platelet aggregation inhibitors were assessed.

### Iron deficiency and anemia definitions

Different clinical definitions of ID were applied to the study cohort, as shown in **Table 1.**

Anemia was defined by hemoglobin levels below 120g/l for women and below 130g/l for men and categorized as IDA, AI, or a combination of AI and IDA, as shown in **Table 2.**

### Statistical analysis

Mean comparison of normally distributed numeric data was performed using Student's *t*-test. If Gaussian distribution was not given, the Mann-Whitney-U-test and Kruskal-Wallis-test were applied. Baseline characteristics in terms of categorical variables were compared using Chi-Square, and Fisher's exact test, where appropriate. Spearman rank correlation technique was used for analysis of monotonic associations in non-normally distributed data. If Gaussian distribution based on Shapiro-Wilk test and a linear relationship were given, Pearson correlation coefficient was calculated to assess the degree of correlation. Trends over time analyses were conducted with paired Student's *t*-test, Wilcoxon test, or Nemar's test, as appropriate. All tests were two-sided and a *p*-value of less than 0.05 indicated statistical significance. A post-hoc power calculation determined a power of 0.92 for the detection of ID assuming a prevalence of ID among COPD patients of 20% according to previous data 21. Statistical analyses were performed with the SPSS 24.0 statistical package (IBM Corp., Armonk, NY, USA).

### Ethical approval

All procedures performed in the present study involving human participants were in accordance with the ethical standards of the Institutional and/or National Research Committee and with the 1964 Helsinki declaration and its later amendments and were performed after approval of the Ethics Committee of the Medical University of Innsbruck (EK Nr.1147/2017).

## Results

### Demographics and iron-metabolism of the study cohort

The study population included 134 (65.7 %) male and 70 (34.3 %) female patients, the mean age was 64.80 ± 10.16 years and the mean follow-up period was 36.83 ± 14.70 months. Patients were allocated to the four COPD stages based on their FEV1 capacity, as defined by the GOLD classification 19. Baseline characteristics, as well as follow-up data of the study population, are shown in **Table 3.**

**Figure 2:**
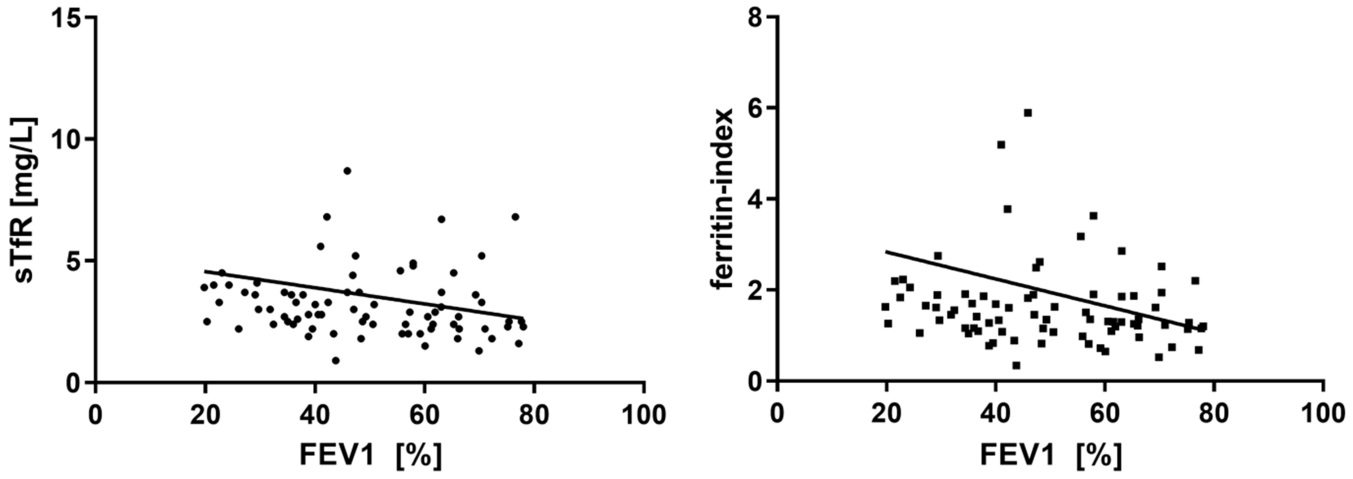
Correlation between FEV1(%) and sTfR ferritin-index.

During follow-up, significant decreases in FEV1 (*p<*0.01) and DLCOc (*p<*0.01) over time were observed in the entire cohort. At least one AECOPD requiring in-hospital treatment was reported from 11 (22.4 %) patients during the observation period. No significant differences in the frequency of ID or in absolute iron parameters were found when comparing patients with and without exacerbations. **Table 3** shows the respective iron parameters at both time-points. Ferritin was the only iron parameter to show a significant change during follow-up with decreasing means (*p=*0.02, **Table 3**). When looking at the correlations between FEV1 and iron parameters at study inclusion, a significant, inverse relationship could be found for sTfR (*r=*-0.27, *p=*0.02, **Figure 2**), and ferritin-index (*r=*-0.23, *p=*0.04, **Figure 2**), whereas no significant correlations were seen between FEV1 and serum iron, ferritin, Tf, and TSAT. CAT-score did not correlate with iron metabolism at study inclusion, nor did FEV1 or CAT-score at follow-up. In contrast, most of the iron parameters revealed a significant correlation with DLCOc at study inclusion (serum iron *r=*0.21, *p<*0.01; Tf *r=*-0.172. *p=*0.03; TSAT *r=*0.246, *p<*0.01; sTfR *r=*-0.341, *p<*0.01; ferritin-index *r=*-0.309, *p=*0.01).

### Prevalence and characterization of ID and Anemia

**Figure 3:**
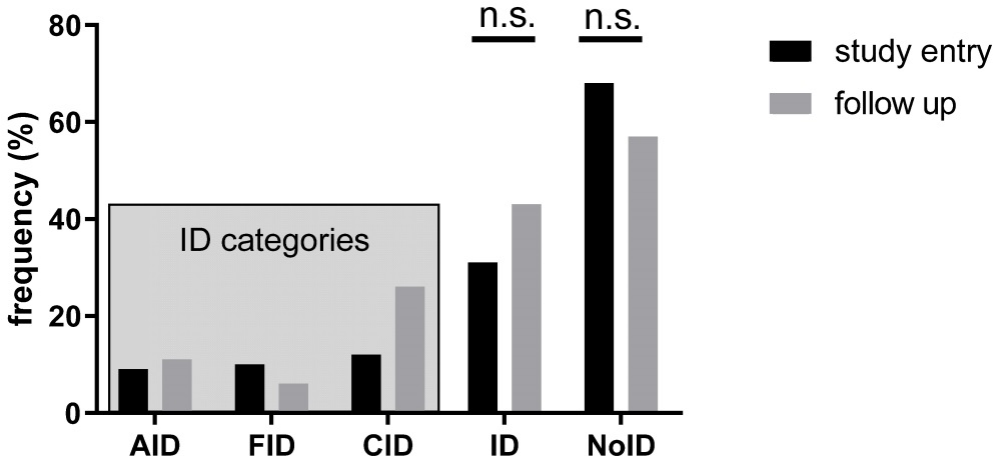
** Prevalence of ID at study inclusion and follow-up.** AID: absolute iron deficiency, FID: functional iron deficiency, CID: combined iron deficiency, ID: iron deficiency, NoID: no iron deficiency, n.s.: not significant at *p* > 0.05.

In our study population, 139 patients (68.1%) had no ID, while 25 patients (12.3%) revealed CID, 21 patients (10.3%) FID and 19 patients (9.3%) AID (**Figure 3**). The absolute number of patients with ID increased during follow-up; however, the observed changes between the two time points were not significant (*p>*0.05) (**Figure 3**).

**Figure 4:**
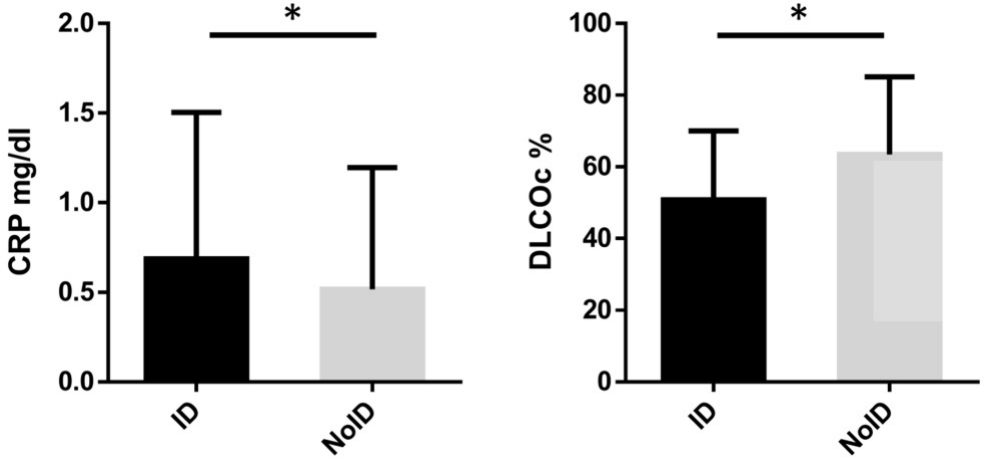
** Diffusion capacity and CRP levels depending on iron status.** * Indicates statistical significance at *p* < 0.05. ID: iron deficiency, NoID: no iron deficiency, DLCOc: diffusion capacity, CRP: C-reactive protein.

Iron-replete patients had a significantly higher DLCOc as compared to patients with ID (63.47 ± 21.64 vs 50.80 ± 19.25 %, *p<*0.01), while CRP (0.52 ± 0.68 vs 0.69 ± 0.82 mg/dl, *p=*0.03) values were significantly lower (**Figure 4**). There were no significant differences in age, FEV1, CAT-score and LVEF when comparing ID with iron-replete patients.

**Figure 5:**
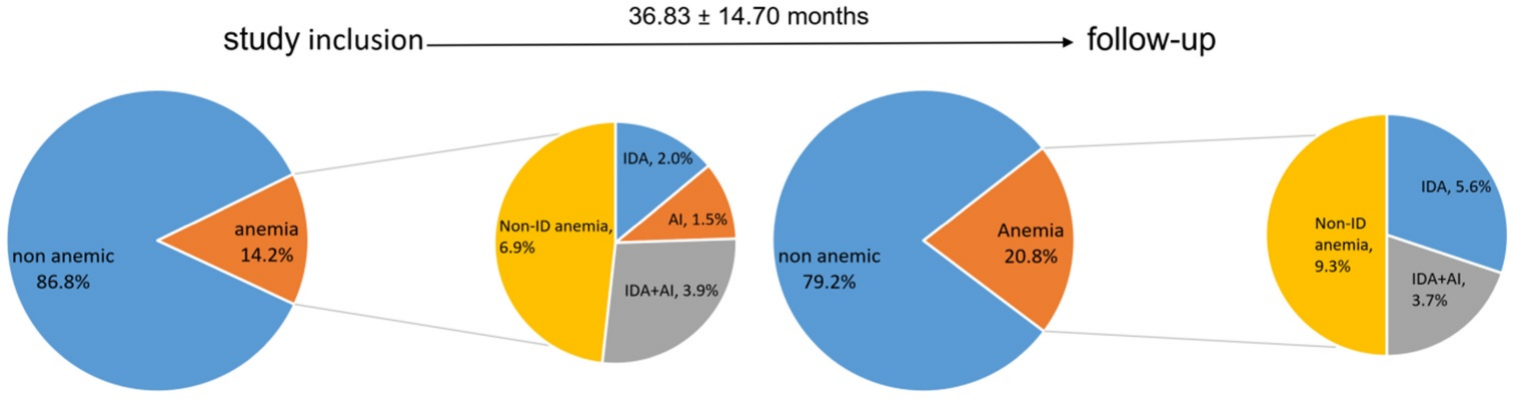
** Distribution of anemia, IDA and ACD at study inclusion, and follow-up.** IDA: iron deficient anemia, ACD: anemia of chronic disease, ID: iron deficiency.

The mean hemoglobin levels at study inclusion were 142.80 ± 16.93 g/L and showed a slight yet not significant decrease during follow up (139.44 ± 17.22g/L, *p=*0.2). Anemia was present in 29 patients (14.2%) at study inclusion, and the prevalence increased to 20.8% during follow-up (*p=*0.5, **Figure 5**). **Figure 5** shows the distribution of anemia types, IDA, IDA + AI and anemia not related to ID at baseline and follow-up. Patients with anemia had a significantly lower FEV1 (43.19 ± 17.57 vs 51.49 ± 17.09%, *p=*0.03) and DLCOc (48.35 ± 17.74 vs 61.12 ± 21.78%, *p=*0.01), as well as higher CRP levels (0.77 ± 0.55 vs 0.54 ± 0.75 mg/dl, *p<*0.01) compared to subjects without anemia irrespective of the presence of ID. CRP levels correlated with ferritin (*r=*0.02, *p<*0.01), Tf (*r=*-0.220, *p<*0.01), and TSAT (*r=*-0.281, *p<*0.001).

A total of 116 patients (56.95 %) received an ACT. No significant differences in Hb, serum iron, ferritin, Tf and TSAT were observed between patients having ACT compared to patients without ACT, whereas sTfR (3.70 ± 2.58 vs. 3.46 ± 0.95 mg/L, *p=*0.02) and ferritin-index (2.23 ± 2.52 vs. 2.00 ± 0.94, *p<*0.01) were slightly, yet significantly higher in patients with ACT. Further, the presence of anemia did not show a significant association with ACT (*p=*0.42) either. A total of 54 (26.5 %) of patients had a gastrointestinal comorbidity. No significant differences in Hb, serum iron, Tf, TSAT or sTfR were detected between patients with or without a gastrointestinal condition.

## Discussion

This study reveals a high prevalence of ID in a real-life COPD cohort, but exact characterization depends on the applied definition. ID is associated with anemia, an elevated inflammatory state, and impaired functional lung capacity, as reflected by FEV1 and DLCOc.

The present study tried to categorize different types of ID, according to commonly applied, robust and easily reproducible ID-definitions. The combined prevalence of all types of ID detected in this study was 30%, the most frequent type being CID. A few studies exploring the possible causality between COPD pathogenesis and severity to ID and/or anemia have already been published 21-27, but the comparison of ID frequencies is difficult as definitions vary across the available literature. Nickol et al. were the first ones to investigate ID using definitions targeting absolute ID and identifying 18% to be iron-deficient 21, which is distinctly more than 9.3% of AID in our cohort. FID was previously described in small observational studies using more liberal definitions, therefore not allowing a direct comparison of prevalence rates 25, 28. The unambiguous definition of ID is crucial, as therapeutic supplementation could prove beneficial only in selected patients. Anemic patients and those with established AID benefit from iron supplementation. In FID, on the other hand, its therapeutic effect may be less, as the route of the problem is elevated hepcidin levels. Iron supplementation will most likely not improve iron homeostasis due to the chronic inflammatory state upregulating endogenous hepcidin production 29.

Further, this study is the first to elucidate the course of ID and anemia in COPD over a follow-up period. The observed significant decrease in ferritin and increase of combined ID are mainly driven by chronic inflammatory processes along with blood losses 15-22. Thus, ID is frequent in COPD and frequently aggravates during the disease. However, as there is no consensus on the exact definition of ID in literature, its estimated prevalence in COPD appears highly variable.

Anemia was present in 14% of patients at study inclusion, and the prevalence slightly increased during follow-up. Anemia was associated with elevated CRP and a lower FEV1, parameters reflecting a more advanced disease, whereas ACT or gastrointestinal comorbidities did not correlate with anemia. This underlines the current evidence that also anemia is highly prevalent among COPD patients, ranging anywhere between 4.9 and 38.0% 22, 30, 31. Anemia of chronic disease is the most common type of anemia in COPD 32 described in the literature, equivalent to this study's findings. The small patient numbers in both studies limit the validity of these results, though. In addition, as ACT did not affect hemoglobin count, its use may be considered safe in this study cohort. Contrasting, a multicenter study of patients with atrial fibrillation, of whom 70% received an ACT, comparing clinical outcomes in subgroups with and without comorbid COPD revealed a higher incidence of hemorrhagic events in COPD patients 33. Concerning the relationship between iron parameters and COPD, only sTfR and ferritin-index were negatively associated with FEV1. Parameters like sTfR or ferritin-index allow more accurate characterization of ID as cellular iron demand, iron storage, and systemic inflammation are taken into consideration. COPD stages, the frequency of severe exacerbations, or symptoms assessed as CAT-Score were not related to systemic ID, which is surprising and questions the role of elevated ID prevalence in the pathogenesis of COPD. In this context, it is essential to distinguish between local and systemic iron dyshomeostasis. Previous studies showed high iron content in lung tissues of advanced COPD patients compared to healthy controls, with a heterogeneous distribution of iron at a cellular level, including mitochondria 34. Mitochondrial iron loading has previously been linked to COPD, as it is upregulated by iron-responsive element-binding protein 2, which is increased in COPD. Its deficiency seems to alleviate cigarette-smoke induced pulmonary inflammation 35 because mitochondrial iron loading impairs mitochondrial function and oxidative phosphorylation 36. Moreover, Yoshida et al. showed that cigarette smoke exposure induces ferroptosis, an iron-dependent form of regulated cell death, which may play a role in COPD pathogenesis 9. Therefore, unbiased correction of ID of any cause may improve systemic iron homeostasis but could have undesired different loco-regional effects in the lung 11. This study highlights that randomized prospective trials investigating the benefit of iron supplementation in COPD are warranted to improve the clinical management of these patients.

Although our data expand the current knowledge about ID in COPD patients, we have to acknowledge some limitations, the major one being the retrospective design. Thus, the observation period was mainly based on the availability of laboratory studies. Also, about 2/3 of the patients were lost during follow-up, biasing the results, especially in terms of anemia, given the low prevalence, especially in absolute numbers. Further, the potential role of nutrition and pulmonary cachexia contributing to ID could not be addressed due to the retrospective study design. Further prospective studies, including organ-specific iron assessment as well as interventional studies investigating the benefit of iron supplementation, are warranted to confirm the herein presented hypotheses.

## Conclusion

ID is common among COPD patients, but the prevalence highly depends on the definition applied, and a uniform definition for accurate diagnosis does not exist. ID is most frequently caused by a combination of absolute and functional ID, and prevalence is increasing during the course of the disease. ID associates with FEV1 and DLCOc restriction, especially when using more robust parameters like sTfR and ferritin-index, which account for inflammation. Respiratory physicians need to be aware of the high prevalence, as it might reflect a more severe COPD phenotype. Prior to iron supplementation, the underlying type of ID should be identified and especially with the presence of AID extended screening to rule out occult blood losses needs to be considered. Proper detection and treatment options of ID need to be evaluated in future prospective studies.

## Figures and Tables

**Table 1:** ID definitions (11)

Definition	Ferritin (μg/L)	TSAT (%)
Absolute ID (AID)	≤ 30	≤ 20
Functional ID (FID)	≥ 100	≤ 20
Combined ID (CID) *	30 - 100	≤ 20
No ID	> 30	> 20

*In five cases the constellation of ferritin < 30 ug/L and TSAT > 20% with Tf being in the normal range was categorized as combined ID.

**Table 2:** Anemia definitions based on ferritin, TSAT, and CRP levels (11)

Definition	Ferritin (μg/L)	TSAT (%)	CRP (mg/dL)
Iron deficiency anemia (IDA)	≤ 30		≤ 0.5
Anemia of inflammation (AI)	≥ 100	≤ 20	> 0.5
Combined (CID = AI + IDA)	< 100		> 0.5

**Table 3:** Baseline characteristics of the study population, at study inclusion, and during follow-up

Parameter		n	Study inclusion	n	Follow-up	*p*-value
Follow-up (months)	36.83±14.70					
Age (years)		204	64.80 ± 10.164	54	66.37 ± 10.164	
**COPD stages#**		204				
Gold 1			8 (3.9%)			
Gold 2			87 (42.6%)			
Gold 3			63 (30.9%)			
Gold 4			46 (22.5%)			
FEV1 (%)		204	50.32 ± 17.37	50	48.36 ± 19.47	< 0.01
DLCOc		166	59.42 ± 21.68	44	55.25 ± 22.80	< 0.01
CAT Score*		146	15.34 ± 8.11	34	18.67 ± 16.72	0.98
ACT^#^		204	116 (56.9%)			
GI comorbidity^#^		204	54 (26.5%)			
AECOPD^#^		49	11 (22.4%)			
**Laboratory parameters**	**Ref. range**					
serum iron (µmol/L)	5.8-34.5	204	16.92 ± 7.08	54	20.14 ± 33.77	0.18
ferritin (µg/L)	30-400	204	152.58 ± 152.67	54	140.13 ± 259.47	0.02
Tf (mg/dL)	200-360	204	257.75 ± 42.62	54	261.86 ± 48.43	0.36
TSAT (%)	16-45	204	26.84 ± 11.90	54	28.72 ± 35.34	0.31
sTfR (mg/L)	<4,5/<5	78	3.59 ± 3.60	39	3.66 ± 2.34	0.18
ferritin index ^(1)^	<3.2/< 2	78	1.97 ± 3.18	39	2.26 ± 2.28	0.18
hemoglobin (g/L)	120-157	204	142.80 ± 16.93	52	139.44 ± 17.22	0.2
MCH (pg)	27.0-32.0	204	30.65 ± 2.52	53	29.69 ± 4.43	0.1
MCV (fl)	77.0-96.0	204	89.50 ± 6.00	53	88.69 ± 9.48	0.3
MCHC (g/dL)	310-360	204	342.32 ± 13.09	53	331.89 ± 36.83	0.16
CRP (mg/dL)	0.00-0.50	204	0.57 ± 0.73	53	0.85 ± 1.30	0.42
creatinine (mg/dL)	0.67-1.17	203	1.06 ± 0.56	54	1.06 ± 0.40	0.19

Quantitative variables are shown as mean ± standard deviation; #parameters are represented as total n and percentage;^ (1)^ ferritin index was calculated as (sTFR/log Ferritin).

## Abbreviations

ACT: Anticoagulation therapy
AI: anemia of inflammation
AID: absolute iron deficiency
CAT: COPD assessment test
CID: combined iron deficiency
COPD: chronic obstructive pulmonary disease
DLCOc: diffusion capacity for carbon monoxide
FEV1: forced expiratory volume in one second
FID: functional iron deficiency
FVC: forced vital capacity
GOLD: Global Initiative for Chronic Obstructive Lung Diseas
ID: iron deficiency
IDA: iron deficiency anemia
LVEF: left ventricular ejection fraction
sTfR: soluble transferrin receptor
